# X-Linked Hypohidrotic Ectodermal Dysplasia (XLHED): A Case Report and Overview of the Diagnosis and Multidisciplinary Modality Treatments

**DOI:** 10.7759/cureus.40383

**Published:** 2023-06-13

**Authors:** Hammad Aftab, Ivan A Escudero, Fatin Sahhar

**Affiliations:** 1 Department of Family Medicine, Detroit Medical Center-DMC/Michigan State University College of Medicine, Detroit, USA

**Keywords:** rare disease, ei200, eda1, eda, xlhed, x-linked, clouston syndrome, christ-siemens-touraine syndrome, ectodermal dysplasia, x-linked hypohidrotic ectodermal dysplasia

## Abstract

Hypohidrotic ectodermal dysplasia (HED) is a rare genetic disorder caused by a mutation in either the ectodysplasin (EDA), ectodysplasin A receptor (EDAR), EDAR associated via death domain (EDARADD), or Wnt family member 10A (WNT10A) genes that result in impaired development of ectodermal-derived structures. The literature defines two types of ectodermal dysplasia, which are hypohidrotic and hidrotic. X‐linked hypohidrotic ectodermal dysplasia (XLHED), also known as Christ-Siemens-Touraine syndrome, is the most common form and is a variant of ectodermal dysplasia characterized by a classical triad of hypo/adontia, hypohidrosis, and hypotrichosis; whereas, hidrotic type of ectodermal dysplasia, also known as Clouston syndrome, is characterized by a triad of onychodysplasia, hypotrichosis, and palmoplantar hyperkeratosis while sparing the sweat glands.

Symptoms of XLHED can begin early in life between the ages of one month to 23 months. XLHED is more commonly seen in males due to the x-linked characteristics of the gene mutations. This disease can be diagnosed by physical exam alone, or in combination with molecular genetic testing. XLHED specifically has an estimated occurrence of one in every 20,000 newborns worldwide. Approximately 5,000 people in the United States have the disease.

In this case report, we present an adult patient diagnosed with XLHED. Our objective is to emphasize the significance of early diagnosis, advocate for a multidisciplinary management approach, and shed light on the potential of recombinant protein and targeted gene therapy for further research. By raising awareness of this condition, we aim to improve patient outcomes not only in newborns but also in adults who have already been diagnosed with XLHED.

## Introduction

Hypohidrotic ectodermal dysplasia (HED) is a rare genetic disorder caused by either a single gene mutation or multiple gene mutations, particularly in the ectodysplasin (EDA), ectodysplasin A receptor (EDAR), EDAR associated via death domain (EDARADD), or Wnt family member 10A (WNT10A) genes [1]. EDA-related HED is inherited in an X-linked manner. On the other hand, HED caused by mutations in EDAR, EDARADD, or WNT10A genes follows an inheritance pattern that can be either autosomal recessive or autosomal dominant. Males with x‐linked hypohidrotic ectodermal dysplasia (XLHED) are hemizygous for EDA gene abnormalities and usually express the full phenotypic characteristics of HED. Females with XLHED are generally heterozygous for an EDA gene abnormality and present with a phenotype ranging from mild HED with hypodontia to the severe form seen in hemizygous males [2]. 

The EDA gene is located on locus Xq12-q13.1 and is responsible for the formation of ectodysplasin A (EDA1) protein; a key protein necessary for connecting ectodermal tissue with mesodermal tissue [1,3]. This assists in the development of ectoderm-derived structures including hair, teeth, skin, and eccrine sweat glands [3]. The EDAR gene is responsible for the formation of ectodysplasin A receptor that interacts with ectodysplasin A protein, leading to a cascade for cell division and growth [4]. EDARADD gene is responsible for creating EDARADD protein that interacts with ectodysplasin A receptor for programmed cell death to assist in specific cell division, growth, and maturation of ectodermal structures [4]. The WNT10A gene is responsible for the formation of WNT10A protein that helps create and shape teeth [5]. Disruptions in the EDA gene can cause the inactivation of EDA1, leading to impaired development of ectodermal placode formation. This, in turn, can result in a range of fetal phenotypic malformations including sparse body hair, tooth agenesis, hypodontia, or abnormal teeth, hoarse voice, cough, dry eyes, dry nasal secretions, dry mucous membranes, and reduced or absent sweat glands [6].

The symptoms of XLHED can vary in severity, ranging from mild to extreme. While some literature sources have proposed logical and potential solutions for XLHED, it is important to note that these are not yet established as the standard of care due to the rarity of the disease. Further research and clinical evidence are needed to validate and establish effective treatment approaches for XLHED.

## Case presentation

**Figure 1:**
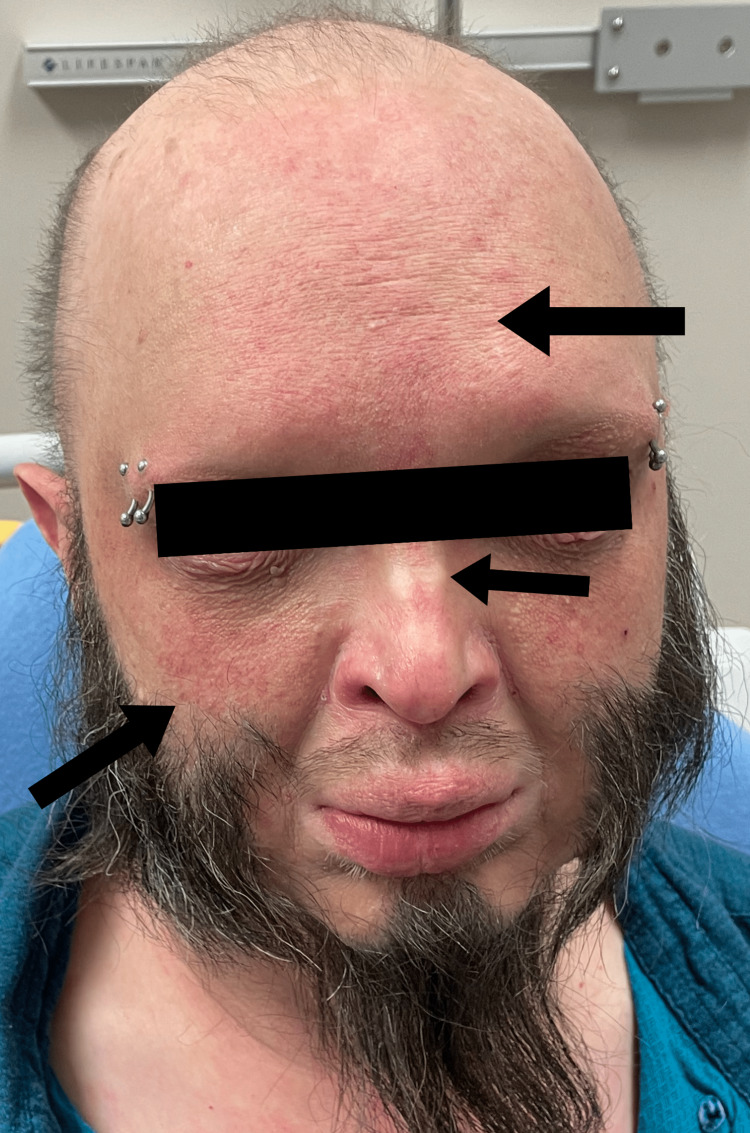
Prominent forehead, eczematous rash on cheek and forehead, and flattened nasal bridge.

**Figure 2:**
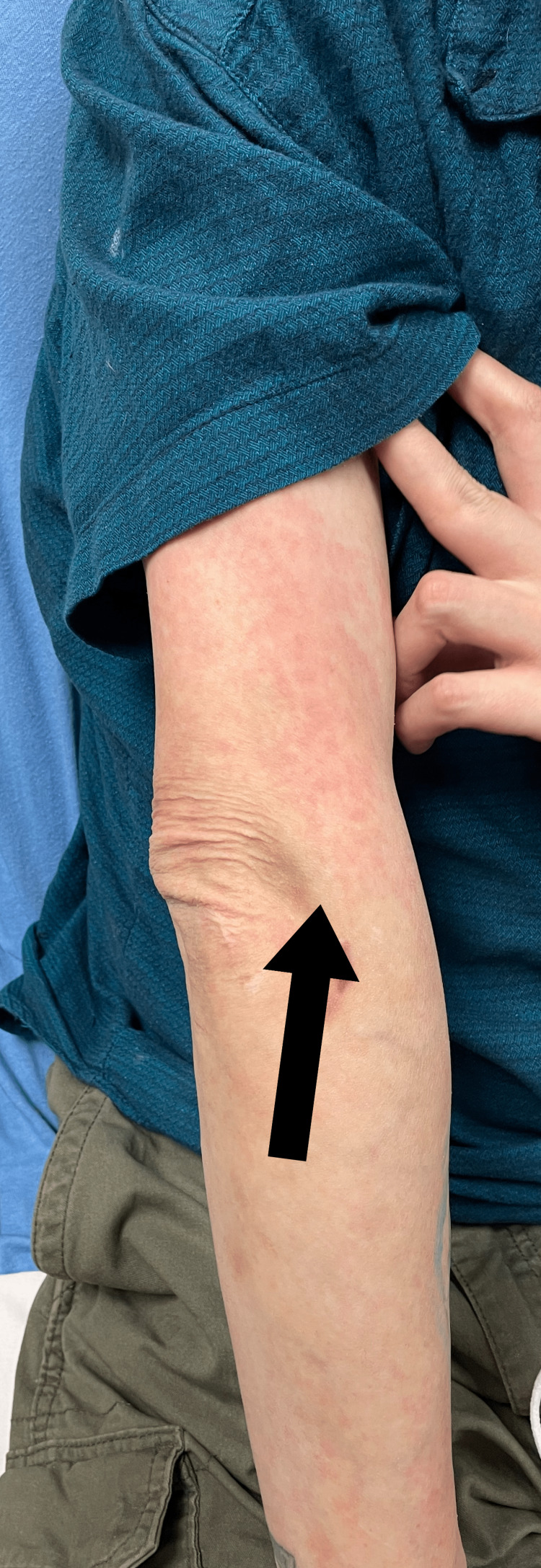
Eczematous rash present on arm.

**Figure 3:**
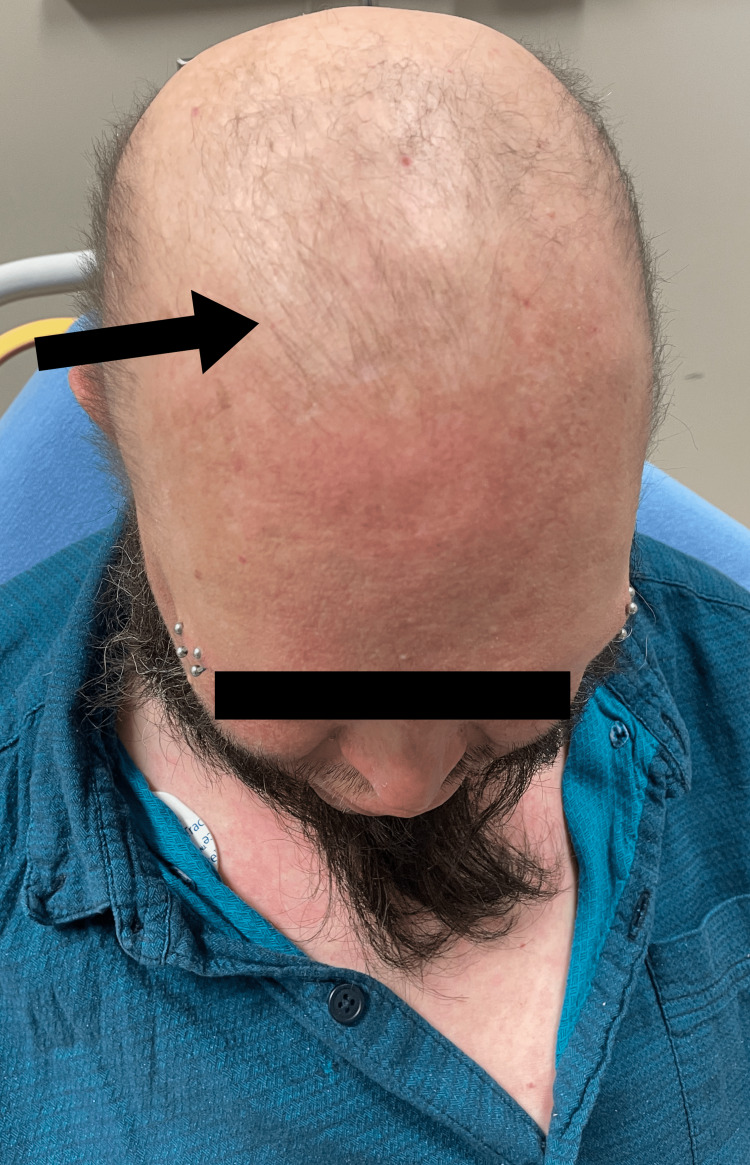
Thin and lightly pigmented scalp hair.

**Figure 4:**
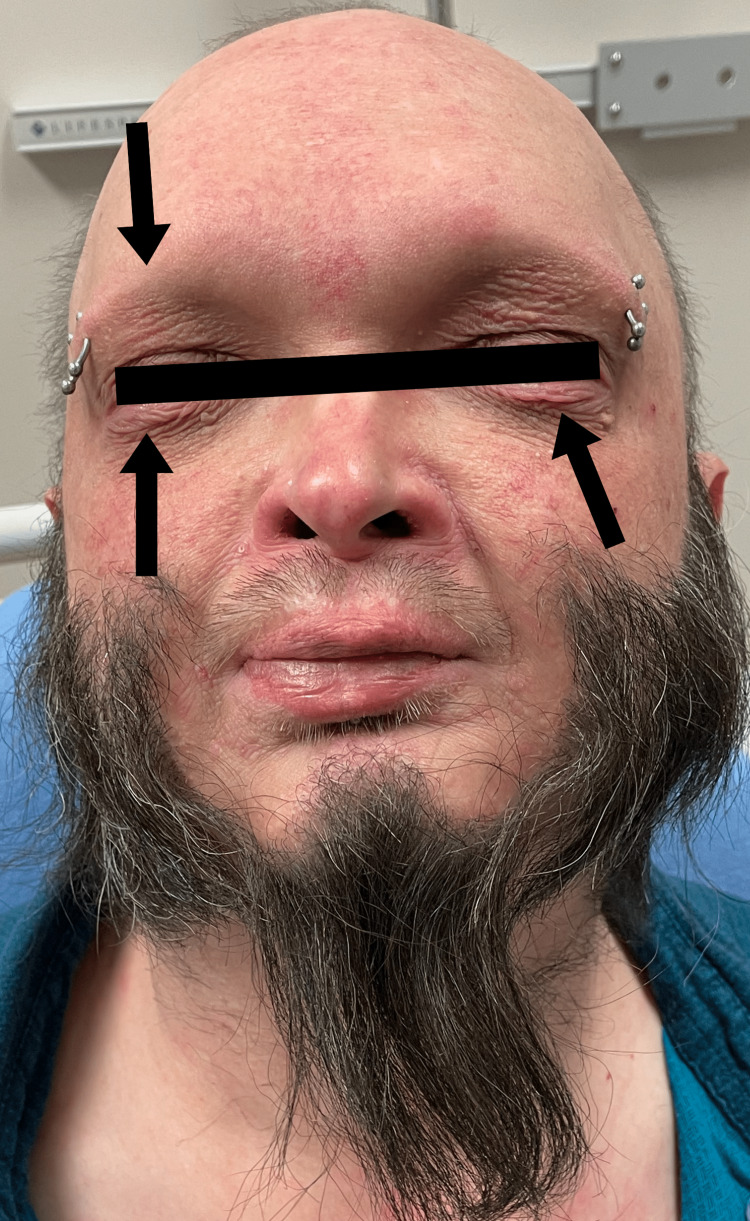
Absence of eyelashes and eyebrows; wrinkling of eyelids.

**Figure 5:**
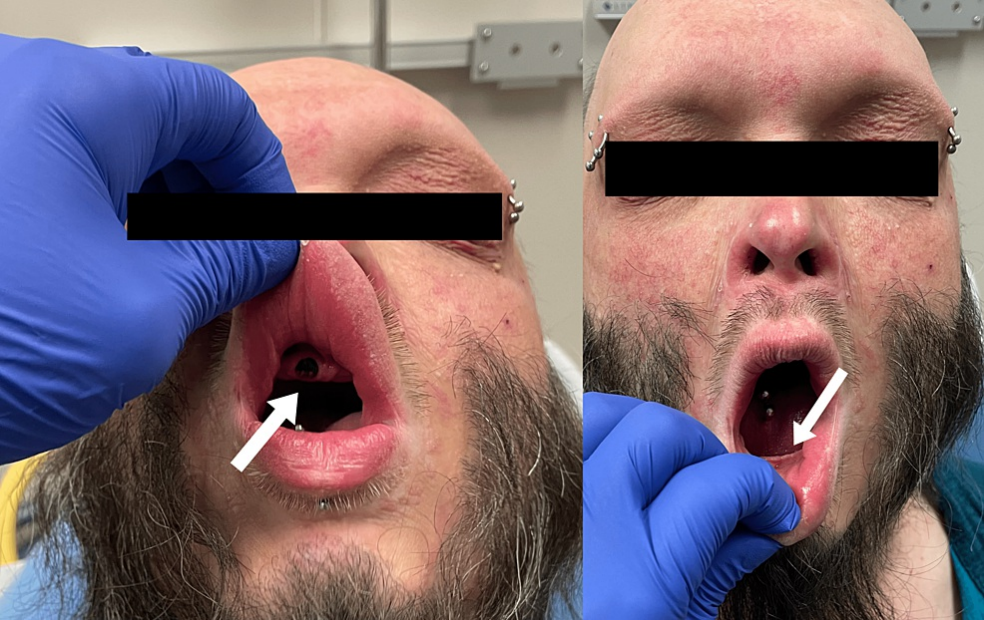
Absence of teeth; the patient had only one tooth, located in his upper gum.

A 44-year-old Caucasian male with multiple co-morbidities of chronic disease presented to the Sinai-Grace Hospital emergency department in Detroit, Michigan via ambulance with complaints of dizziness, palpitations, and anxiety for one day. Although the patient was treated for his dizziness, palpitations, and anxiety, his physical examination revealed a high likelihood of XLHED. Upon further questioning, the patient endorsed chronic cough, persistent respiratory infections, dry skin, dry mouth, absence of teeth, and inability to sweat. The patient stated that he was diagnosed with XLHED at birth by his pediatrician based on his morphological features and family history. He was very familiar with his disease. According to the patient, his hometown in Seminary, Mississippi, where he was born, has a high prevalence of the disease. The patient mentioned that he has a positive family history of XLHED, in which both his mother and brother are positive for the disease. The patient denied any previous genetic testing for the disease. He stated that his symptoms of chronic cough, skin dryness, inability to sweat, and dry mouth have been mostly treated symptomatically since a young age and never followed with a specific specialist. The patient also endorsed recurrent respiratory infections and a raspy voice. He stated that he was diagnosed with chronic obstructive pulmonary disease (COPD) several years ago because of the frequency of difficulty in breathing, chronic cough, and frequent respiratory infections. He also smoked about 1-1.5 packs of cigarettes a day. However, he denied ever undergoing any pulmonary function testing. The patient denied chest pain, shortness of breath, headaches, seizures, abdominal pain, nausea, vomiting, diarrhea, fever, and chills. Physical examination revealed a prominent forehead, flattened nasal bridge, absent eyelashes and eyebrows, wrinkled peri-orbital eyelids, eczematous rash on the cheek, forehead, and arms, thin lightly pigmented scalp hair, chest and bilateral arms, several missing teeth, and dry mucous membranes (Figures 1-5).

Upon the patient's arrival at the emergency department, the initial focus was on addressing his concerns of palpitations and anxiety. Once his condition stabilized, a referral to a dermatologist was provided to address his eczematous rashes, considering that he had not been prescribed any medication or cream for the dermatitis. Additionally, the patient was advised to establish regular biannual follow-ups with a dentist to assess the potential need for dental implants. Patient was advised to follow up with a Pulmonologist for pulmonary function testing to confirm his diagnosis of COPD or if there was a component of his disease which was playing a part in his pulmonary function. We also facilitated a referral for the patient to establish care with a primary care physician to further manage the various symptoms associated with his condition. The importance of regular follow-ups with specialists and a primary care physician for ongoing symptom management was emphasized, and counseling was provided accordingly.

## Discussion

XLHED is a multisystem genetic disorder that affects structures originating from the ectoderm [7]. EDA1 is a transmembrane protein involved in the TNFα-related signaling pathway that assists with the connection of ectoderm to the mesenchyme. This protein is crucial for the formation of hair, teeth, skin, and eccrine sweat glands [8]. Any disruption to EDA1 protein or EDA receptors can manifest in dysmorphic features previously noted. 

Hypohidrotic ED may be inherited as X-linked, autosomal dominant, and autosomal recessive patterns. XLHED is the most common form of the disease [9]. Early diagnosis can allow for timely intervention, appropriate treatment, and support [10]. 

Clinical evaluation

XLHED is characterized by several distinct features. These include generalized hypotrichosis (reduced body hair), thin and sparse scalp hair with light pigmentation, facial dysmorphism (such as frontal bossing and periorbital wrinkling), dental abnormalities like hypodontia, anodontia, or peg-shaped teeth, atopic dermatitis affecting the face and body, absent or reduced sweating, hoarse voice, chronic cough, gastroesophageal reflux, dry eyes, dry nasal secretions, chronic sinusitis, generalized dry skin, and a saddle nose deformity [11]. Although patients generally have sparse body hair and thinning of the scalp hairs, a perplexing finding is that secondary sexual hair including facial hair, axillary hair, and pubic hair can be normal [11]. The reason for sparing of facial, axillary, and pubic hair remains largely unknown. Additionally, the nails are not affected either. Moreover, the aforementioned features are more likely to be seen in males; in females, there may be variability in phenotypic expression because of the carrier state [10-12].

Molecular genetic testing can be performed to identify mutations in the EDA, EDAR, EDARADD, or WNT10A genes, which are responsible for HED. Molecular genetic testing can not only confirm the diagnosis but also identify carriers within the family and serve as a tool for genetic counseling.

Differential diagnosis

When considering a differential diagnosis for XLHED, it is important to take into account other forms of HED with different inheritance patterns such as autosomal recessive or autosomal dominant HED. These conditions may exhibit similar symptoms but have distinct genetic inheritance patterns.

Another condition to consider is ectrodactyly-ectodermal dysplasia-clefting (EEC) syndrome, characterized by missing fingers or toes (ectrodactyly), ectodermal dysplasia, and cleft lip/palate. While EEC syndrome shares some features with XLHED, the presence of clefting and ectrodactyly can help differentiate between the two conditions. 

Ichthyosis is a diagnosis to consider in patients who present with skin changes such as dry, scaly skin with varying scales. XLHED is associated with hypohidrosis, while ichthyosis typically does not affect sweat gland function. XLHED may involve teeth and hair abnormalities, as well as eye abnormalities, whereas ichthyosis primarily affects the skin without tooth or hair abnormalities.

Furthermore, acquired causes of hypohidrosis such as certain medications, nerve damage, or autoimmune diseases can lead to reduced sweating and may resemble some symptoms of XLHED. However, these conditions typically lack the characteristic genetic inheritance pattern associated with XLHED.

Treatment modalities and management

Dental care: Early dental interventions focus on addressing the dental abnormalities associated with XLHED. This may involve bonding of conical teeth, dental prosthetics, such as dentures or bridges, to replace missing teeth, orthodontic treatment, and regular dental check-ups every six-twelve months to monitor tooth development and manage dental caries [12]. As such there is no definitive time for starting dental treatment; however. it is suggested that initial prosthesis should be provided before the patient begins school [13]. Frequent drinking of water and use of fluoride supplementation would be necessary to avoid the formation of dental caries [14]. Additionally, dietary counseling for chewing difficulties may be warranted, as well as speech language therapy for patients with swallowing difficulties [11].

Dermatological care: The management of dry skin and atopic dermatitis in XLHED involves the use of moisturizers, emollients, and topical therapies to alleviate symptoms and maintain proper skin health. Regular skin care routines, including gentle cleansing, are recommended just as with any dry skin-related disorders. Use of sunscreen is also recommended. Referral to a dermatologist may also benefit the patient [11].

Pulmonological/ENT care: Counseling for cessation of smoking should be provided at every office visit to reduce the risk of respiratory infections. Humidification of air, frequent use of nasal saline spray, and lubrication eye drops may also assist in relieving dryness [15]. Referral to a pulmonologist, otolaryngologist, or allergist may also be beneficial [11].

Heat intolerance management: Individuals with XLHED have reduced or absent sweat glands that can lead to heat intolerance or hyperthermia [16]. Heat mitigation strategies may include the use of cooling devices, avoiding excessive heat exposure, staying hydrated, wearing lightweight, breathable clothing, and implementing measures to maintain a cool environment that can reduce the risk of overheating [16]. Artificial sweat substitutes in the form of sprays or gels can be used to compensate for reduced or absent sweating. These substitutes help regulate body temperature and prevent overheating.

Pregnancy management: Pregnant patients with XLHED are classified as high-risk. Optimal prenatal nutrition, use of prenatal vitamins, and avoidance of situations that can increase the risk of hyperthermia should be discussed at every antenatal visit. Referral to OB/GYN may also be considered [11].

Genetic counseling: Genetic counseling is essential for individuals with XLHED and their families. It provides information on inheritance patterns, recurrence risks, reproductive options, and available support networks [11].

Psychological and emotional support: Living with a rare genetic disorder can present emotional and psychological challenges. Supportive counseling and access to patient support groups can help individuals and families cope with the emotional impact of XLHED and provide a platform for sharing experiences and advice [17].

So far, there are only supportive measures for treating XLHED focusing on improving quality of life [11]. However, there are a few studies that have demonstrated the prevention and reversal of XLHED with promising results. Margolis et al. stated that previous studies using dog models demonstrated partial prevention of the XLHED phenotype by way of ultrasound-guided prenatal administration of recombinant EDA1, called EDI200. Their research has shown that there were improved phenotypic outcomes including growth rates, lacrimation, hair growth, meibomian gland formation, sweating, dentition, and mucociliary clearance. Essentially, all dog animals treated prenatally with recombinant EDA1 showed positive responses compared with untreated dogs with XLHED [18].

Hermes et al. have attempted intra-amniotic administration of EDI200 in pre-natal mice and found phenotypically different mice after birth. They found that postnatal mice had visible phenotypic changes including dense coats, normal hair distribution around the eyes, normal shaped tails, dark spots on paws, and normal eye opening. They also performed histological evaluations of the mice and confirmed the presence of eccrine glands [19].

Schneider et al. used recombinant EDA1 prenatally in six boys. They used an EDA1 replacement protein and injected the protein into the amniotic fluid, which showed to consistently induce the development of functional sweat glands. Normal ability to sweat has so far persisted for >five years in the boys treated in utero. Thus, timely replacement of a missing protein appears to be a promising therapeutic strategy for the most frequent ectodermal dysplasia and possibly additional congenital disorders [20]. Although prenatal administration of Fc-EDA shows promise, its effectiveness on permanent teeth development seems to be suboptimal at present. Additionally, no corrective effects have been observed on the development of scalp hair and primary teeth, both of which are ectodermal structures formed during early embryogenesis [21].

It was noted that there were no detectable immune responses to the investigational drug in infants who had received Fc-EDA in utero. The safety profile of this drug encourages further development of prenatal EDA1 replacement therapy [22]. Intra-amniotic administration of recombinant EDA1 protein can potentially reverse XLHED, which is currently being investigated in a multicenter clinical trial [20]. It is worth noting that the development and testing of pharmacological interventions for XLHED are still ongoing, and these treatments are not yet approved or widely available.

The patient described in this case report experienced numerous challenges related to symptoms that he has been coping with since childhood. Unfortunately, he did not receive adequate counseling or close monitoring from primary care or specialized healthcare providers. This lack of support potentially contributed to the worsening of his symptoms, including recurrent respiratory tract infections resulting from ongoing smoking versus his disease process, as well as heightened anxiety due to managing a rare disorder. These circumstances highlight the significance of regular follow-ups with specialists and the importance of ongoing counseling for patients diagnosed with XLHED.

## Conclusions

This case report highlights the importance of understanding the clinical characteristics, diagnostic challenges, and multidisciplinary approach to managing XLHED. Early recognition, comprehensive supportive care, and genetic counseling play a vital role in enhancing the quality of life for individuals affected by XLHED.

Further comprehensive research is needed to explore additional treatment modalities for XLHED due to its impact on multiple ectodermal structures. While genetic therapies using recombinant proteins have shown promise in neonates, further follow-up studies with larger participant numbers are necessary before considering it as a preferred in utero treatment option. There is optimistic potential that this treatment approach could benefit mothers who are carriers of the genetic disease. However, currently, there is no specific treatment available for adults who have already been diagnosed with XLHED, except for supportive measures. More research is required to investigate the feasibility and efficacy of utilizing recombinant proteins and genetic therapies for adolescents and adults with XLHED. Raising awareness of this rare condition serves as a catalyst for continued research and the advancement of targeted gene therapy approaches. However, in the absence of a definitive cure, personalized care plans remain crucial in effectively managing the disease.
